# Analgesia Is Enhanced by Providing Information regarding Good Outcomes Associated with an Odor: Placebo Effects in Aromatherapy?

**DOI:** 10.1155/2013/921802

**Published:** 2013-06-10

**Authors:** Yuri Masaoka, Miho Takayama, Hiroyoshi Yajima, Akiko Kawase, Nobuari Takakura, Ikuo Homma

**Affiliations:** ^1^Department of Acupuncture and Moxibustion, Faculty of Health Sciences, Tokyo Ariake University of Medical and Health Sciences, 2-9-1 Ariake, Koto-ku, Tokyo 135-0063, Japan; ^2^Department of Physiology, Showa University School of Medicine, 1-5-8 Hatanodai, Shinagawa-ku, Tokyo 142-8555, Japan; ^3^Japan School of Acupuncture, Moxibustion, and Physiotherapy, 20-1 Sakuragaoka-cho, Shibuya-ku, Tokyo 150-0031, Japan; ^4^The Foundation for Oriental Medicine Research, 28-9 Sakuragaoka-cho, Shibuya-ku, Tokyo 150-0031, Japan

## Abstract

No previous report has described whether information regarding an odor used in aromatherapy has placebo effects. We investigated whether placebo analgesia was engendered by verbal information regarding the analgesic effects of an odor. Twelve of 24 subjects were provided with the information that a lavender odor would reduce pain (informed), whereas the other 12 subjects were not (not-informed). Concurrent with respiration recording, the subjects were administered a lavender-odor or no-odor treatment during application of painful stimulation to the forefinger. The subjects reported their experience of pain and its unpleasantness on a visual analogue scale after the painful stimulation. The lavender-odor treatment significantly alleviated pain and unpleasantness compared with the no-odor treatment in the informed (*P* < 0.01) and not-informed groups (*P* < 0.05). The no-odor treatment in the informed group significantly alleviated pain and unpleasantness compared with both the no-odor and lavender-odor treatments in the not-informed group (*P* < 0.05). Rapid and shallow breathing induced by the painful stimulation became slow and deep during the lavender-odor and no-odor treatments in both groups. Information regarding a lavender odor, the lavender odor itself, and slower breathing contributed to reduced perceptions of pain and unpleasantness during painful stimulation, suggesting that placebo effects significantly contribute to analgesia in aromatherapy.

## 1. Introduction

Application of aromatherapy has shown recent growth as a complementary and alternative medicine for pain alleviation, stress management, relaxation in daily life, and enhancement of meditation in clinical practice [1, 2]. Particularly in clinical applications, aromatherapy has been used to reduce pain and accompanying unpleasantness [3–5]. Although studies show that aromatherapy is quite effective in alleviating pain, little is known regarding the mechanisms underlying this effect [3–5], especially any placebo effects of aromatherapy.

A number of factors may be involved in the analgesic effects of aromatherapy. The odor itself may alleviate pain through changes in brain activity, especially in pain-related regions. Olfaction is a unique sensory process as odor information directly ascends to the piriform cortex, the entorhinal cortex (which is a gateway to the hippocampus), and the amygdala without passing the thalamus [6]. The limbic system, including the amygdala, is well known to be an area having pain-related processes [7]. The direct input of odor information to limbic areas may contribute to the modification of pain sensations and unpleasantness.

Another factor in the analgesic effects of aromatherapy is changes in respiratory patterns with odor stimulation. Because odor perception is largely dependent on inspiration, every inspiration delivers odor molecules to the receptors of the olfactory nerve and activates olfactory limbic areas. Breathing patterns are changed unconsciously by the stimulation of the limbic system. Pleasant odors increase tidal volume (*V*_*T*_) and decrease respiratory frequency, thereby producing slow and deep breathing. Induction of slow and deep breathing patterns by aromatherapy may be one mechanism by which it reduces pain sensations [8].

Another factor in the analgesic effects of aromatherapy may be placebo effects unconsciously engendered by aromatherapy, which may have relevance for which odors to use in aromatherapy. The placebo effect is defined as “the nonspecific psychological or psychophysiological therapeutic effect produced by a substance or procedure that is without any therapeutic effect for the specific condition being treated.” [9]. For example, if a subject is given a sugar pill and told that the pill potently reduces pain, the actual experience of pain relief in the subject represents the placebo effect. In aromatherapy or clinical trials, aromatherapists or researchers often provide patients with descriptions such as “this odor has great effects for stress reduction,” “this odor may reduce your anxiety and allow you to sleep easier,” and “this odor will mitigate your pain sensations.” Such descriptions may influence subjective emotional states and alter the effectiveness of aromatherapy or the outcomes of clinical trials. To the best of our knowledge, however, the role of placebo effects in aromatherapy has not been investigated.

In this study, we investigated whether and to what extent placebo effects contributed to analgesic effect engendered by aromatherapy, in comparison to the effects of the odor itself and the effects of changes in respiration.

## 2. Method

### 2.1. Subjects

Twenty-four subjects (mean age 30 ± 9 years, fourteen men and ten women) participated in this study. All subjects provided written informed consent, and the study was approved by the Ethics Committee of the Showa University School of Medicine.

### 2.2. Olfactory Acuity Test

Prior to the experiments, odor detection and recognition acuities in every subject were evaluated by means of the T&T olfaction test (Takasuna Co., Ltd., Tokyo, Japan). Five odors (A, *β*-phenyl ethyl alcohol, B, methyl cyclopentenolone, C, isovaleric acid, D, *γ*-undecalactone, and E, skatole) were tested using an olfactometer. Each odorant dissolved in propylene glycol was presented at eight different concentrations. In each subject, we applied each odorant starting at the lowest concentration and presented higher concentrations in ascending order. During each test, the subject was asked whether an odor was detected. Following detection of an odor, the subject was required to identify and describe the odor. The concentration at which the odor was perceived but not identified was considered the detection level. The concentration at which an odor was first identified was considered the recognition level. Each subject's odor detection threshold was expressed as the average of all odor threshold scores (A + B + C + D + E/5). Olfactory detection and recognition levels in all subjects were confirmed to be within normal ranges (mean threshold level, −0.7 ± 0.2, mean recognition level, 0.2 ± 0.3) according to a prior study [10].

### 2.3. Electrical Stimulation to Induce Pain

Subjects were seated on a chair and two electrodes were attached to the dorsal side of the distal part of the right forefinger to apply the painful stimulation. Stimulation was delivered using electrical currents with a 1 ms duration and 1 s interval that were generated by a commercially available stimulator (MEB-4204, Nihon Kohden, Tokyo, Japan) through an isolator (NM-430S, Nihon Kohden).

First, we determined the perception threshold of each subject to the electrical stimulation. Then, subjects were administered a stimulation at intensities below the perception threshold to observe respiratory responses under the no-pain condition.

Second, the pain threshold of each subject to the electrical stimulation was determined. The pain threshold was described by a current at which each subject began to perceive pain. Then, we set the voltage of the electrical stimulation to 110–115% of the pain threshold of each subject to induce easily detectable pain. The mean and standard deviation (SD) of the current required to induce easily detectable pain across all subjects were 14.3 ± 4.6 mA.

In the following experiments, five sets of the electrical stimuli (at intensities below the perception threshold or the threshold to induce easily detectable pain) for 30 s with a 30 s interval were delivered to the subjects. We used this duration because it minimizes olfactory habituation to the odor stimulation [11] presented during the painful stimulations.

### 2.4. Pain Measurement

After each painful stimulation, the intensity of the painful stimulus and its unpleasantness was measured on a 100 mm visual analogue scale (VAS). The right edge of this VAS was “most imaginable pain” or “most imaginable unpleasantness” and the left edge was “no pain” or “no unpleasantness.”

### 2.5. Lavender-Odor and No-Odor Treatments

We used a lavender odor (Pranarom, France) in this study, as in one of our previous studies [12] and no-odorant litmus strips for the no-odor treatments. For the odor presentation, lavender oil was diluted to a concentration of 30% with ethanol and applied to litmus strips. Lavender-odorant-dipped litmus strips were prepared 30 minutes before the experiment and left exposed to the air until the smell of alcohol dissipated. The odorant-dipped litmus strip or the no-odorant litmus strip was presented in front of the inspiratory side of a one-way valve connected to a transducer. When the subject inspired, the inspiratory valve opened until the onset of expiration, when the expiratory valve opened. Odorants were inspired through the transducer, which measured the respiratory data.

To confirm the perceptions and emotions induced by the odor, the level of pleasantness of the odor was measured after each trial using a separate VAS that consisted of a 200 mm horizontal line. The maximum rating on the right edge was “most imaginable pleasantness,” the middle point was “neither pleasant nor unpleasant,” and the maximum rating on the left edge was “most imaginable unpleasantness.”

### 2.6. Respiratory Measurement

A facemask with a transducer connected to a respiratory flow monitor (Minato Medical Science, Osaka, Japan) was attached to each subject for the measurement of respiratory patterns. The flow monitor was connected to a Power Lab respiration recorder (AD instrument, Tokyo, Japan). Offline analyses were performed by a Power Lab Scope (AD instrument), and we calculated breath-by-breath *V*_*T*_ and total respiratory time (*T*_tot_).

### 2.7. Experimental Procedure

**Figure 1:**
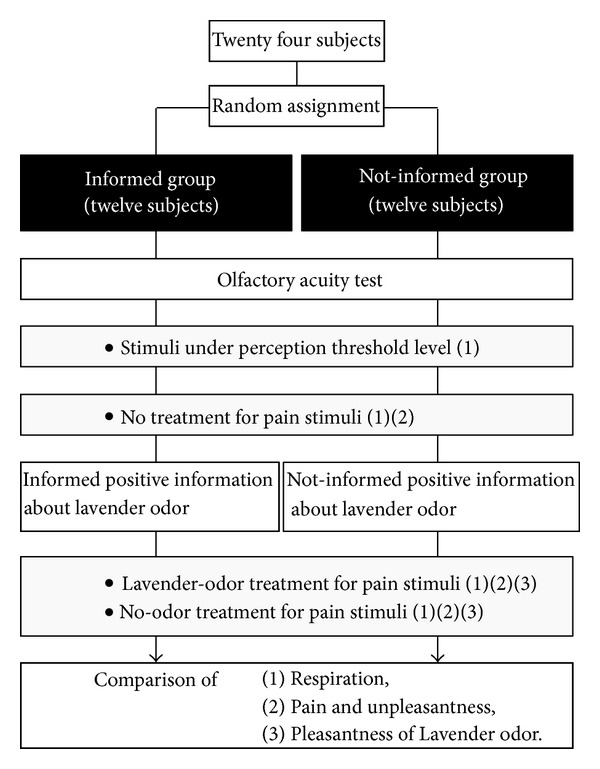
Description of the experimental procedures. The recording of respiration (1), measurement of pain and unpleasantness (2), and measurement of the perceived pleasantness of the lavender-odor or the no-odor (3) is illustrated.

Figure 1 shows an illustration of the experimental procedures. The 24 subjects were initially randomly divided into two groups of twelve subjects, with seven males and five females in each group. Then, we confirmed that olfactory detection and recognition levels in all subject were within the normal range as evaluated by an olfactory acuity test. Two experimenters conducted the experiments, one examiner who delivered electrical stimuli at intensities below the thresholds for perception or pain and another who applied odor. The experimenter who delivered the electrical stimuli was asked to not talk with the subjects and to convey the same attitude with every subject. The experimenter who delivered the odor held the same conversation and expressed the same attitude toward the subjects in the informed and not-informed groups except for providing the following positive information about the lavender odor to the subjects of the Informed group.

All subjects in both groups received five sets of electrical stimuli at intensities below their perception threshold defined as a result of prior testing for 30 s with a 30 s interval between each stimulus. We recorded respiration during this stimulation below the level of perception (no-pain condition).

Next, the subjects received five sets of painful stimulations each separated by a 30 s interval to obtain control data (no treatment). We recorded respiration during each set of painful stimuli and measured the intensities of pain and unpleasantness on the VAS after each set of painful stimulations in every subject. Then, the subjects in one group were provided with descriptions of the effects of the odor (informed, see later), whereas those in the other group were not (not-informed).

For the informed group, the descriptions of the effects of the odor presented were the following, “It has been reported that pleasant odors reduce pain and its unpleasantness. A lavender odor is particularly effective in alleviating pain. The effects of the lavender odor are (1) to improve the quality of sleep; (2) to ease pain and its unpleasantness; and (3) to help body and mind relax.” We also explained that “There are two example studies that show the lavender odor pronouncedly reduces anxiety [4] and pain sensation [5] after surgery. There are many reports of the lavender odor being used as meditation in clinical field.” The subjects in the not-informed group were not provided with the descriptions and were simply told that the odor would be delivered sometime during the experiment.

In each group, we applied five sets of the painful stimulations that were randomly assigned to the lavender-odor or no-odor treatment. We recorded respiration during each painful stimulation and measured the intensities of pain and unpleasantness and the level of pleasantness of the odor on the VAS after each painful stimulation in every subject.

### 2.8. Data Analysis

All statistical analyses were performed with a commercially available statistical package (SPSS, Ver.11.0; SPSS, Tokyo, Japan). For each subject, changes in pain and unpleasantness scores engendered by the lavender-odor or no-odor treatment were obtained by subtraction of the pain and unpleasantness scores during no treatment from those scores during presentation of the lavender-odor and no-odor treatment, respectively. The mean and standard error of the mean (SEM) for the changes in pain and pleasantness were calculated in each group. We analyzed the changes in the pain and unpleasantness among the four groups (lavender-odor and no-odor treatments in the informed group and lavender-odor and no-odor treatment in the not-informed group) by one-way analysis of variance (ANOVA). Post hoc tests between the four groups were performed using the *Scheffe *test. Using the same analytic methods, changes in the pleasantness scores elicited by the lavender-odor treatment were also evaluated.

The *T*_tot_ and *V*_*T*_ values were compared between the no-pain condition, no treatment (the painful stimulation alone) condition, and the conditions of painful stimulation with the lavender-odor or no-odor treatment in the informed group and the painful stimulation with the lavender-odor or no-odor treatment in the not-informed group by one-way ANOVA and *Scheffe *tests.

Pearson correlation coefficients for the linear regression between respiratory responses and changes in pain/unpleasantness scores were calculated across all of the subjects and for each group.

## 3. Results

**Figure 2:**
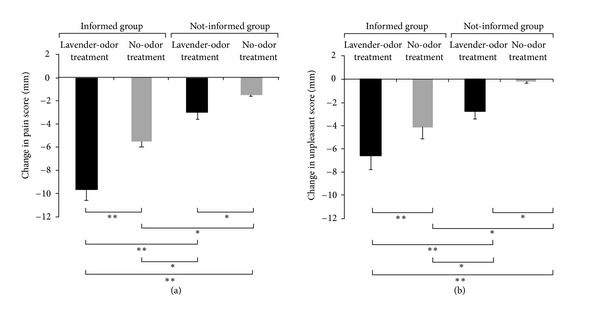
Changes in pain (a) and unpleasantness (b) scores during the lavender-odor and no-odor treatments in the informed and not-informed groups. For each subject, changes in the pain and unpleasantness scores during the lavender-odor or the no-odor treatments were obtained by subtraction of the pain and unpleasantness scores during no treatment from those scores during the lavender-odor and non-odor treatments, respectively. The changes in the pain and unpleasantness scores in each group are presented as the mean ± standard error (SE). Minus values indicate decreases. *P < 0.05, **P < 0.01.

Figure 2 shows the effects of the lavender-odor or no-odor treatment on the perception of pain (a) and unpleasantness (b) in the informed and not-informed groups. There were significant differences between the four groups in the changes in their pain and unpleasantness scores (*P* < 0.001, resp.).

Post hoc tests showed that the decreases in pain and unpleasantness scores engendered by the lavender-odor treatment were significantly larger than those engendered by the no-odor treatment in the informed (pain and unpleasantness, *P* < 0.01) and not-informed groups (pain and unpleasantness, *P* < 0.05).

Importantly, even with the no-odor treatment in the informed group, pain scores showed larger reductions compared with the lavender-odor treatment in the not-informed group (*P* < 0.05). For the respective decreases in the pain and unpleasantness scores, the lavender-odor and no-odor treatments in the informed group were significantly larger than the lavender-odor (pain, *P* < 0.01, unpleasantness, *P* < 0.01) and no-odor (pain and unpleasantness, *P* < 0.05) treatments in the not-informed group, respectively.

**Figure 3:**
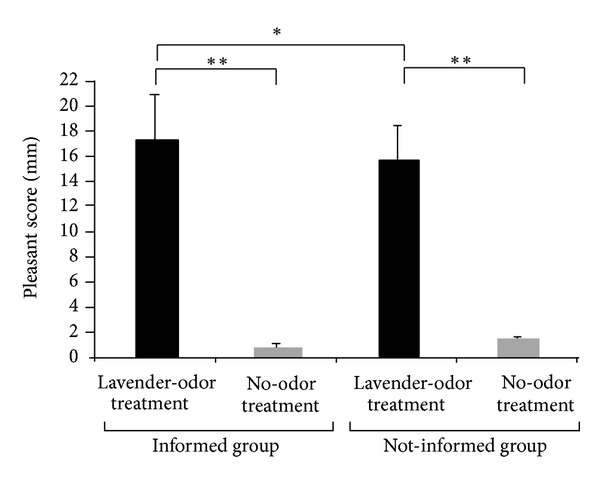
Perceived pleasantness scores for the lavender-odor and no-odor treatments in the informed and not-informed groups. **P < 0.01, *P < 0.05.

We confirmed that the subjects felt pleasant during the lavender-odor treatment and did not feel pleasant during the no-odor treatment (ANOVA, *P* < 0.01, Post hoc tests, *P* < 0.01, resp.) (Figure 3). The subjects in the informed group perceived the lavender-odor treatment as being more pleasant than those in the not-informed group (*P* < 0.05).

**Figure 4:**
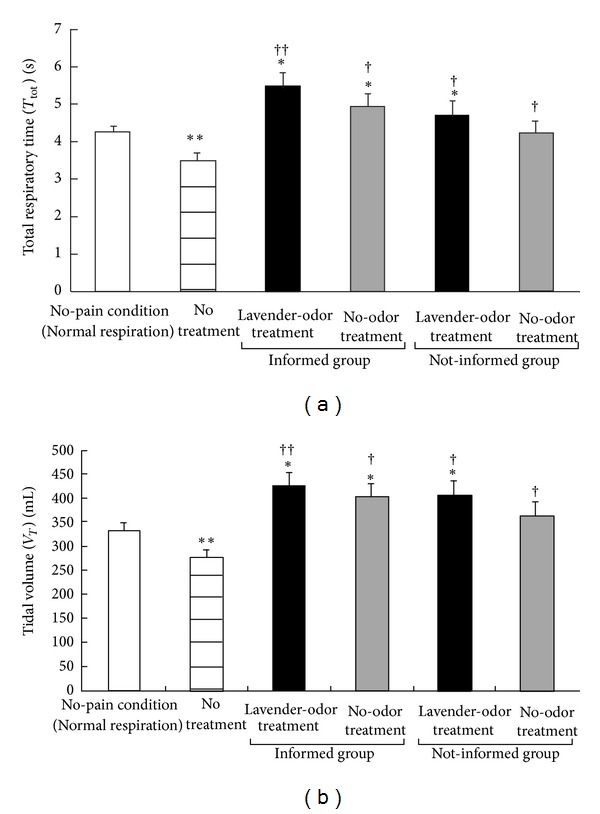
Total respiratory time ((a), *T*_tot_) and tidal volume ((b), *V*_*T*_) during the condition without painful stimulation (no-pain condition), the painful stimulation with no treatment (control), and the painful stimulation with lavender-odor or no-odor treatment in the informed and not-informed groups. *P < 0.05, **P < 0.01, significant difference from the no-pain condition. ^†^ *P* < 0.05, ^††^ *P* < 0.01, significant difference from no treatment.

Figure 4 shows the *T*_tot_ and *V*_*T*_ data during the no-pain condition, no treatment condition, and the conditions of pain stimulation during the lavender-odor or no-odor treatment in the informed groups and the painful stimulation with the lavender-odor or no-odor treatment in the not-informed groups. There were significant differences between the six conditions in *T*_tot_ and *V*_*T*_ (*P* < 0.01, resp.). Post hoc tests showed that *T*_tot_ (*P* < 0.01) and *V*_*T*_ (*P* < 0.01) decreased during painful stimulation compared with no-pain condition. In both the informed and not-informed groups, application of the lavender-odor or the no-odor treatment increased *T*_tot_ and *V*_*T*_ levels compared with no-pain condition (*P* < 0.05 in all conditions), except those of the no-odor treatment in the not-informed groups.

*T*_tot_ and *V*_*T*_ levels increased in every condition compared with no treatment (lavender odor in the informed group, *P* < 0.01, the other conditions, *P* < 0.05).

Association between respiratory responses and changes in pain and unpleasantness scores across all subjects and in each group was examined by calculation of correlation coefficients.  Table 1 shows the correlation coefficients (*r*) and *P* values for all trials. *T*_tot_ and *V*_*T*_ levels during each condition were not associated with changes in pain or unpleasantness.

## 4. Discussion

Using the lavender-odor and no-odor treatments, in this study, we tested whether positive information regarding an odor presented in aromatherapy affects its analgesic effects. Although factors such as (1) induction of deep and slow breathing, (2) the pleasantness of the odor, and (3) placebo effects related to information regarding an odor may be involved in analgesia, the current results suggest that prior information regarding an odor has a greater impact on analgesia than breathing changes or the perceived pleasantness of the odor.

### 4.1. Effects of Slow Breathing on Pain and Unpleasantness Perceptions

Both pain sensations and unpleasantness associated with pain decrease *V*_*T*_ and increase the respiratory rate (RR) [12, 13]. Consistent with these findings, in this study, the painful stimulation shortened *T*_tot_ and reduced *V*_*T*_. That is, it induced rapid and shallow breathing. In contrast, regardless of the informed or not-informed groups, the lavender-odor and no-odor treatment increased *T*_tot_ and *V*_*T*_, which engendered deep and slow breathing. Thus, it could be argued that these changes in respiratory patterns induced by the lavender-odor or the no-odor treatment may have modulated pain and pain-induced emotions. In this regard, our data coincide with previous reports that voluntary slow breathing reduces pain [8] and a decreased RR modifies anxiety [14].

It has been suggested that deep and slow breathing influences the activity of the autonomic nervous system and pain processing [8]. Suppressing arousal of sympathetic activity by deep and slow breathing may modulate pain perception [15]. Also, deep and slow breathing may affect limbic activity, especially that of the amygdala. Importantly, decreasing RRs suppresses the activity of the amygdala [6], which may ease unpleasantness of pain and vice versa. These mechanisms may be involved in the changes in analgesia observed in this study.

Although deep and slow breathing has been applied in various relaxation techniques, changes in breathing may be a minor factor in aromatherapy because our observed increases in the *T*_tot_ and *V*_*T*_ did not differ significantly across the four groups receiving the lavender-odor or the no-odor treatment. This occurred even though the perceptions of pain and unpleasantness did differ significantly across these groups.

### 4.2. Effects of Lavender Odor on Pain and Unpleasantness Perceptions

A previous study reported that pleasant odors reduce pain sensations and unpleasantness, which were associated with slower breathing [12]. Based on the significant differences in pain and unpleasantness between the lavender-odor and no-odor treatments in the informed and not-informed groups, respectively, the lavender odor had specific effects on pain perception, as in a previous study [12]. If there was no expectation in the subjects receiving the no-odor treatment or in the not-informed group, the decrease in pain and unpleasantness could be attributed to deep and slow respiration. Therefore, we presume that the difference in pain between the lavender-odor and no-odor treatments in the not-informed group was engendered by the odor itself. If so, odor information directly ascending to olfactory limbic structures—including the entorhinal cortex, amygdala, and hippocampus—may modulate perceptions of pain and its unpleasantness.

### 4.3. Effects of Expectations on Pain and Unpleasantness Perceptions

In this study, the most prominent decrease in the pain perception and unpleasantness was observed in the subjects receiving the lavender-odor treatment in the informed group. From visual inspection of the differences in the attenuation of pain between the lavender-odor treatments in the informed group and not-informed group, no-odor treatments in the informed group and not-informed group, and no-odor treatments in the informed group and the lavender-odor treatment in the not-informed group, it is likely that positive information regarding the lavender odor engendered analgesic effects equal to, or perhaps greater than, the odor itself. This suggests that the positive information such as “this odor is able to reduce your pain sensation” partially contributes to the analgesic effects of aromatherapy. Indeed, the effects of expectancies can override the pharmacological effects of a drug [16]. This may be the first study to show placebo effects produced by expectancy of the analgesic effects in aromatherapy.

A previous study showed that expectancies reduce stress and pain [17], which may be mediated by activation of the endogenous opiate system that is a part of the descending pain-modulating networks, as is the case with placebo analgesia engendered by cognitive and motivational factors [18]. A previous brain imaging study showed that placebo analgesia was associated with deactivation of the thalamus and the secondary somatosensory cortex, which are pain processing areas. On the contrary, this placebo analgesia was associated with activation of the rostral anterior cingulate cortex, anterior insula, and ventral striatum [19]. The ventral striatum and rostral anterior cingulate cortex are regions related to reward and expectation processing. The activation of these brain regions may modulate pain perception by deactivating pain processing areas [19]. We infer that expectation verbally induced by the positive information regarding the lavender odor may modulate such pain-related processing systems and thereby contribute to placebo analgesia in aromatherapy.

Our results may have a link to cognitive behavior therapy (CBT), which holds that cognition influences emotions and behavior, and this model has been applied to using various tasks as a treatment for people with high anxiety and depression [20]. People having such symptom may also choose aromatherapy, which can be an effective treatment for managing their symptoms. Aromatherapy shares common aspects with CBT by changing cognition through verbal or visual descriptions associated with an odor. It may be important to note that the relationship between emotion and cognition is bidirectional such that changes in emotion can alter cognition. This view may also be important for interpreting our results. 

Although expectations derived from the positive information regarding the lavender odor seem to be another main driver of analgesia in our study, it is worth noting that the difference in pain between the lavender-odor and no-odor treatments in the informed group was obviously larger than that between these treatments in the not-informed group. This suggests that analgesic effects of the lavender odor were supported by the positive information regarding the lavender odor treatment. Additionally, the positive information was more effective when it was presented in combination with the lavender odor. From these findings, we infer that there potentially are mutually reinforcing analgesic effects of the lavender odor and the expectations produced by positive information.

As we mentioned, olfactory information directly ascends to limbic systems such as the amygdala and hippocampus. These regions are the purported center of emotions and are involved in processing pleasure/reward expectations [21]. The extensive overlap in the neural circuitry of olfactory and reward expectation processing may support the mutual reinforcement between lavender odors and expectations produced by positive information. Furthermore, pleasant sensation from lavender odors also may support analgesia because such limbic regions are implicated in pleasure processing.

Excitation by olfactory stimuli transmits to conscious processing of olfactory perception in the orbitofrontal cortex after subconscious processing in olfactory limbic areas [22]. The influence of higher cognitive functions such as word [23] and face recognition processing by dynamic subconscious activity in limbic systems by odor stimulation [24] could be another candidate mechanism for the mutual reinforcement of analgesia by the lavender odor and expectations related to positive information. Collectively, the odor, expectations, and pleasant sensations may all enhance the analgesic effects of aromatherapy.

It cannot be ignored that conditioning, which is another main candidate mechanism for the induction of placebo effects, may have been involved in the placebo effects measured in this study [25]. Conditioning theory suggests that the placebo response is a form of classical conditioning that is based on learning through association [26], such that a rat receiving a paired electrical shock (unconditioned stimulus) and odor (conditioned stimulus) can exhibit a conditioned fear response to the odor [26]. Moreover, patients regularly taking analgesics can experience pain relief when given placebo pills of a similar shape, color, or taste to the analgesics [27]. However, most of the subjects in this study reported that they had no previous experience with aromatherapy using pleasant odors for relaxation. Even if the placebo effect in this study was mediated by a combination of expectancy and conditioning, the involvement of conditioning may be less robust.

It may have significantly impacted the placebo analgesia produced in this study that the subjects were provided indications of positive outcomes by lavender odor in the verbal descriptions. It is probable that descriptions of negative outcomes would have engendered unfavorable outcomes (nocebo effect) with aromatherapy. In future research, factors such as how prior information affects sensations and emotions, how these effects differ across odors (e.g., favorable or nonfavorable odors), and different placebo effects in individuals should be examined to elucidate the placebo effects of aromatherapy.

### 4.4. Limitations

In this study, the examiners were not blinded to the lavender-odor and no-odor treatments. The subjects may have been sensitive to the examiner's facial expressions, the examiner's way of talking, or the examiner's tone of voice [28–30]. Although we paid careful attention to provide the same attitude and voice tone to all subjects and tried to standardize the positive description of the odor, the subjects may have been sensitive to expectations subtly implied by the examiner [31]. Unblinded examiners or providers become strong placebo generators, and thus, placebo effects attributable to the examiners cannot be excluded from the current findings. In future studies of aromatherapy, provider masking should be performed, as in other fields of complementary and alternative medicine [32], in combination with improved patient blinding to the odor treatments.

## 5. Conclusions

Information regarding a lavender odor, the lavender odor itself, and slower breathing contributed to reduced perceptions of pain and unpleasantness during painful stimulation, suggesting that placebo effects significantly contribute to analgesia in aromatherapy.

## Figures and Tables

**Table 1:** Correlations between respiratory variables and pain/unpleasant scores.

		All subjects (*N* = 24)		All subjects (*N* = 24)		Informed group (*N* = 12)				Not-informed group (*N* = 12)			
		Lavender-odor treatment		No-odor treatment		Lavender-odor treatment		No-odor treatment		Lavender-odor treatment		No-odor treatment	
		Pain	Unp	Pain	Unp	Pain	Unp	Pain	Unp	Pain	Unp	Pain	Unp
*T*_tot_	*r*	−0.38	−0.17	−0.12	−0.28	−0.35	−0.01	0.04	−0.11	0.03	0.11	0.37	0.03
	*P*	0.06	0.42	0.55	0.17	0.25	0.76	0.91	0.71	0.91	0.72	0.23	0.92

*V*_*T*_	*r*	0.08	−0.15	−0.05	−0.08	0.25	−0.19	0.18	−0.12	0.19	0.12	0.2	0.23
	*P*	0.68	0.48	0.79	0.69	0.43	0.54	0.57	0.71	0.54	0.71	0.52	0.47

*T*_tot_: total respiratory time, *V*_*T*_: tidal volume, Pain: changes in pain scores, Unp: changes in unpleasant scores, *r*: correlation coefficient, and *P*: *P *value.
